# Validation of Textbook Outcome in Gastric Surgery (TOGS) for Primary Gastric Cancer in an Eastern High-Volume Center

**DOI:** 10.1245/s10434-026-19522-3

**Published:** 2026-04-03

**Authors:** Ludovico Carbone, Yo-Seok Cho, Min Kyu Kang, Kyoyoung Park, Jane Chungyoon Kim, Sa-Hong Kim, Jeesun Kim, Nina Rebecca Kalaw, Yoonjin Kwak, Hye Seung Lee, Seong-Ho Kong, Do Joong Park, Daniele Marrelli, Han-Kwang Yang, Franco Roviello, Hyuk-Joon Lee

**Affiliations:** 1 Division of Gastrointestinal Surgery, Department of Surgery, Seoul National University Hospital, Seoul, Republic of Korea; 2 Department of Medicine Surgery and Neuroscience, University of Siena, Siena, Italy; 3 Department of Surgery, Seoul National University College of Medicine (SNUCM), Seoul, Republic of Korea; 4 Department of Surgery, Cardinal Santos Medical Center, Manila, Philippines; 5 Department of Pathology, Seoul National University Hospital, Seoul, Republic of Korea; 6 Department of Pathology, Seoul National University College of Medicine (SNUCM), Seoul, Republic of Korea; 7 Cancer Research Institute, Seoul National University College of Medicine (SNUCM), Seoul, Republic of Korea; 8 Department of Oncology, Azienda Ospedaliero Universitaria Senese (AOUS), Siena, Italy

**Keywords:** Textbook outcome in gastric surgery, Gastric cancer, Gastrectomy, Validation, Hospital stay, Survival

## Abstract

**Background:**

Textbook Outcome defines the ideal perioperative course after surgery. A specific Textbook Outcome in Gastric Surgery (TOGS) was developed in Western centers; however, its validation among Eastern patients has rarely been investigated. We assessed its achievement in a Korean cohort and identified its predictors.

**Methods:**

We included adults who underwent curative distal or total gastrectomy for gastric cancer (GC) between 2013 and 2023. TOGS consisted of three surgical criteria (no intraoperative complications, R0 resection, and adequate lymphadenectomy: > 20 nodes in subtotal and > 25 in total gastrectomy) and four postoperative criteria (no re-intervention, no unplanned intensive care unit stay, no unplanned 90-day readmission or mortality).

**Results:**

Of the 5806 patients with GC enrolled, 4338 (74.7%) achieved the TOGS, with rates of 77.9% for stage I tumors and 68.8% for stages II–III. The TOGS rate declined with age, from 82.3% in patients aged < 40 years to 67.5% in those aged >80 years, with hospital readmission being the strongest negative predictor of its achievement in the elderly. Several factors were independently associated with a higher likelihood of TOGS: early lesions (*P* = 0.003), female sex (*P* < 0.001), recent surgery (*P* = 0.024), distal gastrectomy (*P* < 0.001), and minimally invasive approach (*P* < 0.001). Patients with TOGS had shorter hospital stays (*P* = 0.014) and improved 5-year overall survival (87.6%, vs. 75.6%, *P* < 0.001), independent of the pathological stage (stage I, *P* < 0.001; stages II–III, *P* < 0.001).

**Conclusions:**

The new definition of TOGS provides valuable insights into the quality of surgical care for patients with GC and has a strong impact on oncological outcomes, including in Eastern patients.

**Supplementary Information:**

The online version contains supplementary material available at 10.1245/s10434-026-19522-3.

Gastric cancer (GC) is the fifth most common malignancy worldwide.^1^ Despite a global decline in incidence, mortality rates after gastrectomy still reach 10% in certain registries, and the 5-year overall survival (OS) often fails to exceed 40%.^2^ Eastern countries such as Japan and Korea achieve superior results, largely due to early detection through national screening programs and standardized treatment protocols, whereas Western series often report delayed diagnosis and higher postoperative mortality.^3,4^

These discrepancies emphasize the need for reliable benchmarks to evaluate surgical quality.^5^ Although numerous studies have advocated for the centralization of complex surgical procedures and reported improved outcomes in high-volume hospitals,^6,7^ no consensus exists on defining volume thresholds. Such cutoffs are arbitrarily determined and differ considerably across Eastern and Western contexts, thereby limiting the comparability of institutional performance.^8^

The emerging concept of the Textbook Outcome (TO) addresses previous shortcomings by integrating multiple perioperative indicators into a single composite metric, thus offering a more comprehensive assessment of quality of care.^9–11^ Most evidence suggests that achieving a TO is associated with longer survival after gastrointestinal resections for cancer.^12^ Recently, a definition specific to GC, the TO in Gastric Surgery (TOGS), has been introduced between Western centers. TOGS was associated with lower postoperative mortality rates and shorter hospital stays.^13^ However, its validation in East Asian populations, where clinical practices and disease profiles differ, remains unexplored.

This study aimed to assess the achievement and prognostic significance of TOGS within a large Korean cohort undergoing curative gastrectomy for GC, thereby externally validating its applicability in an Eastern population.

## Methods

A retrospective cohort study was designed including East Asian patients operated on between 2013 and 2023 at the Seoul National University Hospital in South Korea. The study adhered to the Declaration of Helsinki statements and was approved by the institutional review board (number E-2510-025-1681). We followed the STROBE study guideline checklist for the respective article type.

### Population and Measures

We reviewed the electronic medical records of adult patients who underwent gastrectomy for non-metastatic gastric adenocarcinoma with potentially curative intent. Inclusion criteria were total or distal gastrectomy, American Society of Anesthesiologists score 1–3, and elective surgery. Exclusion criteria were previous gastrectomy; tumor involving the gastro-esophageal junction (Siewert 1 and 2); proximal, pylorus-preserving, and wedge gastrectomies; and missing essential data for assessment of both versions of TO.

Data regarding patient demographics, tumor pathology, management, perioperative course, and survival (5 years) were retrospectively collected.

TO was achieved when all 10 (according to the Dutch Upper Gastrointestinal Cancer Audit [DUCA])^14^ and seven (according to the Italian Gastric Cancer Research Group [GIRCG], Siena 2024) short-term parameters were fulfilled (Table 1). The item “Board evaluation” was excluded in the adapted version of TOGS because of significant differences in recommendations across Eastern countries, which arise from a variety of factors such as local incidence rates, disease stages, and government policies.^15^

**Table 1:** Textbook Outcome (TO; Dutch Upper Gastrointestinal Cancer Audit [DUCA]) and Textbook Outcome in Gastric Surgery (TOGS; Italian Gastric Cancer Research Group [GIRCG]) definitions

TO	TOGS	Adapted TOGS for Eastern populations
Curative resection as judged by the surgeon		
No intraoperative complication	No intraoperative complication	No intraoperative complication
Negative resection margins	Negative resection margins	Negative resection margins
> 15 lymph nodes sampled	Adequate lymphadenectomy, > 20 nodes in subtotal and > 25 in total gastrectomy	Adequate lymphadenectomy, as defined in the TOGS
No postoperative complications Clavien–Dindo grade ≥ II		
No reintervention 30 days after surgery	No reintervention 30 days after surgery	No reintervention 30 days after surgery
No unplanned ICU 30 days after surgery	No unplanned ICU 30 days after surgery	No unplanned ICU 30 days after surgery
Length of stay ≤ 21 days		
No unplanned 30-day hospital readmission	No unplanned 90-day hospital readmission	No unplanned 90-day hospital readmission
No 30-day mortality	No 90-day mortality	No 90-day mortality
	Board evaluation (prn, compliance with neoadjuvant therapy)	

ICU Intensive care unit

### Surgical Course

All operations followed the general principles of open, laparoscopic, or robotic gastrectomy and standardized stepwise procedures. Patients were placed under general anesthesia in the supine position. Peritoneal washing cytology was performed in cases with serosal exposure or when peritoneal metastasis was suspected. Partial omentectomy was performed in most gastrectomies; however, in cases of tumors with serosal exposure, a total omentectomy was preferred. Lymph node dissection was carried out according to the recommendations of the Japanese guidelines, specifically D1+ for early GC and D2 for advanced GC.^16^ After transection of the stomach, the proximal and distal resection margins were routinely checked by frozen section biopsy; if margins were not clear, additional resection was performed. In distal gastrectomy, the reconstruction method (Roux-en-Y, Billroth I, or Billroth II) was determined by the surgeon’s judgment or tumor location. Except for a subset of Billroth I cases in which a circular stapler was used, all anastomoses were performed with a linear stapler. In total gastrectomy, Roux-en-Y esophagojejunostomy was consistently performed with a circular stapler, whereas jejunojejunostomy was constructed with a linear stapler using the side-to-side method.

Patients at stage II or III confirmed on final histopathological examination received adjuvant chemotherapy according to the Korean guidelines.^17^

The follow-up protocol consisted of visits every 6 months for early-stage disease during the first 5 years after surgery or every 3 months for advanced-stage disease during the first 2 years, followed by every 4–6 months for the subsequent 3 years.

### Endpoints

The primary objective of this study was to assess our achievement rates of the Western-defined TOGS,^13^ together with temporal trends in surgical outcomes and quality of care. Additional aims included identifying predictive factors associated with TOGS compliance and analyzing its correlation with OS.

### Statistical Analysis

Continuous variables with non-normal distribution are presented as medians with interquartile ranges (IQRs) and compared using the Mann–Whitney U test. Categorical variables are reported as frequencies and proportions and compared between groups using the χ^2^ test or analysis of variance, as appropriate. A two-sided *P*-value < 0.05 was considered statistically significant. Predictive factors for achieving TO and single parameters were assessed using multivariate logistic regression. For discrete covariates with more than two categories, the highest or most common value was selected as the reference (e.g., age, body mass index [BMI]). For non-ordinal categorical variables with more than two levels, the reference category was chosen based on clinical consensus (e.g., year of surgery), established guidelines (e.g., tumor stage), or group size (e.g., surgical approach). Simple and partial (controlling for age and BMI) correlations were used to test relations between TOGS and hospital stay. Survival analyses are conducted using the Kaplan–Meier method and a multivariable Cox proportional hazards model. SPSS version 20.0 (SPSS Inc., Chicago, IL, USA) and GraphPad Prism version 9.5.0 (GraphPad Software, San Diego, CA, USA) were used.

## Results

Of 8037 patients, a total of 5806 who underwent total or distal gastrectomy with curative intent were enrolled in the study. We excluded 358 proximal gastrectomies, 1411 pylorus-preserving and 260 wedge gastrectomies. An additional 202 cases involving palliative surgery were also excluded.

TO was reached in 4404 patients (75.9%) and adapted TOGS in 4338 (74.7%). Demographic, surgical, and histopathological features are shown in Table 2. Female sex, age ≤ 65 years, and minimally invasive distal gastrectomy for medium-lower third tumors were independently associated with higher TOGS rates (*P* < 0.001).

**Table 2:** Patient characteristics

Characteristic	Entire cohort (*n* = 5806)	TO			Adapted TOGS		
		Yes (*n* = 4402)	No (*n* = 1404)	*p*-Value	Yes (*n* = 4338)	No (*n* = 1468)	*p*-Value
*Baseline*							
Sex Female Male	1932 (33.3) 3874 (67.7)	1577 (81.6) 2825 (79.2)	355 (28.4) 1049 (20.8)	**<0.001**	1527 (79.0) 2811 (72.6)	405 (21.0) 1063 (27.4)	**<0.001**
Age, years	63 (52–70)	63 (54–70)	64 (56–71)	**<0.001**	63 (55–71)	65 (57–73)	**<0.001**
BMI, kg/m^2^	24.1 (22.0–26.3)	24.1 (22.0–26.3)	24.2 (22–26.2)	0.785	24.1 (22.0–26.3)	24.2 (22.1–26.3)	0.804
ASA score 1 2 3 x	1352 (23.3) 3830 (66.0) 425 (7.3) 199 (3.4)	1063 (78.6) 2921 (76.3) 175 (41.2) 143 (81.9)	289 (21.4) 909 (23.7) 250 (58.8) 36 (18.1)	**<0.001**	1072 (79.3) 2861 (74.7) 263 (61.9) 142 (71.4)	280 (20.7) 969 (25.3) 162 (38.1) 57 (28.6)	**<0.001**
Tumor location Upper Medium Lower Diffuse	987 (17.0) 1514 (26.1) 3228 (55.6) 77 (1.3)	647 (65.6) 1152 (72.1) 2555 (79.1) 48 (62.3)	340 (34.4) 362 (23.9) 673 (20.8) 29 (37.7)	**<0.001**	653 (67.2) 1158 (76.5) 2478 (76.8) 49 (63.6)	334 (33.8) 356 (23.5) 750 (23.2) 28 (36.4)	**<0.001**
Neoadjuvant chemotherapy	127 (2.2)	98 (77.2)	29 (22.8)	0.719	94 (74.0)	33 (26.0)	0.854
*Surgery*							
Gastrectomy Distal Total	4455 (76.7) 1351 (23.3)	3520 (79.0) 882 (65.3)	935 (21.0) 469 (34.7)	**<0.001**	3458 (77.6) 880 (65.1)	997 (22.4) 471 (34.9)	**<0.001**
Lymphadenectomy D1/D1+ D2 or more	3052 (52.6) 2754 (47.3)	2409 (78.9) 1993 (72.4)	643 (21.1) 761 (27.6)	**<0.001**	2333 (76.4) 2005 (72.8)	719 (23.6) 749 (27.2)	**<0.001**
Multivisceral resection	292 (5.0)	158 (54.1)	134 (45.9)	**<0.001**	170 (58.2)	122 (41.8)	**<0.001**
Surgical approach Open Minimally invasive	1386 (23.9) 4420 (76.1)	852 (42.0) 3550 (75.8)	534 (58.0) 1070 (24.2)	**<0.001**	886 (63.9) 3452 (78.1)	500 (36.1) 968 (21.9)	**<0.001**
Operation time, min	210 (175–250)	205 (170–245)	220 (185–270)	**<0.001**	208 (170–245)	220 (185–265)	**<0.001**
Hospital stay, days	8 (7–10)	7 (7–8)	14 (9–25)	**<0.001**	7 (7–9)	11 (8–22)	**<0.001**
*Tumor*							
Lauren histotype Intestinal Diffuse/mixed Undefined	2688 (46.3) 2725 (46.9) 393 (6.8)	2020 (75.1) 2097 (77.0) 285 (72.5)	668 (24.9) 628 (23.0) 108 (27.5)	0.086	1985 (73.8) 2076 (76.2) 277 (70.5)	703 (26.2) 649 (23.8) 116 (29.5)	**0.019**
Signet ring cells	372 (6.4)	326 (87.6)	46 (12.4)		296 (79.6)	76 (20.4)	
Lymph nodes removed	40 (31–52)	39 (29–50)	38 (29–51)	0.672	39 (31–51)	34 (22–48)	**<0.001**
pT/ypT 1 2 3 4 x	3529 (60.8) 679 (11.7) 896 (15.4) 672 (11.6) 30 (0.5)	2822 (80.0) 505 (74.4) 615 (68.6) 442 (48.0) 18 (60.0)	707 (20.0) 174 (25.6) 281 (31.4) 230 (52.0) 12 (40.0)	**<0.001**	2755 (78.1) 517 (76.1) 605 (67.5) 441 (65.6) 20 (66.7)	774 (21.9) 162 (23.9) 291 (32.5) 321 (34.4) 10 (33.3)	**<0.001**
pN/ypN 0 1 2 3 x	3941 (67.9) 696 (12.0) 557 (9.6) 597 (10.3) 15 (0.2)	3103 (78.7) 508 (73.0) 380 (68.2) 402 (67.3) 9 (60.0)	838 (21.3) 188 (27.0) 177 (31.8) 195 (32.7) 6 (40.0)	**<0.001**	3031 (76.9) 507 (72.8) 386 (70.4) 405 (67.8) 9 (60.0)	910 (23.1) 189 (27.2) 171 (29.6) 192 (32.2) 6 (40.0)	**<0.001**
TNM stage I II IIIA IIIB-IIIC x	3776 (65.0) 975 (16.8) 465 (8.0) 560 (9.6) 30 (0.6)	3002 (79.5) 706 (72.4) 309 (66.5) 367 (65.5) 18 (60.0)	774 (20.5) 269 (27.6) 156 (33.5) 193 (34.5) 12 (40.0)	**<0.001**	2942 (77.9) 693 (71.1) 314 (67.5) 369 (66.9) 20 (66.7)	834 (22.1) 282 (28.9) 151 (32.5) 191 (34.1) 10 (33.3)	**<0.001**

Bold indicates significant results

ASA American society of anesthesiologists scale, BMI Body mass index, TO Textbook outcome as defined by the dutch upper gastrointestinal cancer Audit, TOGS Textbook Outcome in Gastric Surgery as defined by the Italian gastric cancer research group

### Textbook Outcome in Gastric Surgery

Among surgical criteria contained in the TOGS definition,^13^ intraoperative complications occurred in 77 patients (1.3%), negative resection margins in 5775 (99.5%), and adequate lymphadenectomy in 5441 (93.7%). Among postoperative criteria, a reintervention was required for 617 patients (10.6%), surgical in 64 while under local anesthesia in 553 (endoscopic intervention in 183, radiological/percutaneous intervention in 348, and other in 22 patients), unplanned intensive care unit or medium care unit admission in 93 (1.6%), and 90-day readmission and mortality in 592 (10.2%) and 13 (0.2%), respectively. Most non-surgical reinterventions consisted of percutaneous drainage for postoperative collections in 251 patients (4.3%), endoscopic treatment for stenosis in 85 patients (1.5%), and endoscopic management of anastomotic leakage in 80 patients (1.4%). Postoperative bleeding occurred in 27 patients (0.5%), of whom 17 underwent non-invasive reintervention.

**Fig. 1:**
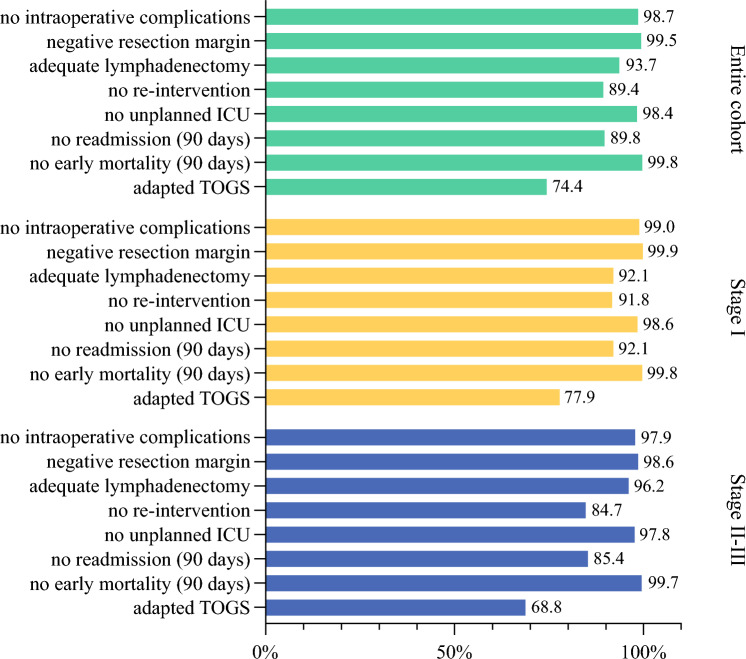
Bar chart rating the rates of Textbook Outcome in Gastric Surgery (TOGS) and its individual parameters

When stratified by pathological stage, TOGS was achieved in 2942 of 3776 stage I tumors (77.9%), in 693 of 975 stage II tumors (71.1%) and 683 of 1025 stage III tumors (66.6%). Within the individual parameters, the absence of reinterventions and readmissions were the most negatively impacting factors for achieving TOGS across all stages, as detailed in Fig. 1. In particular, 323 patients were readmitted within 1 month after surgery and 269 within 3 months. The most frequent causes were nutritional and/or metabolic disturbances and oncologic treatment-related issues, occurring in 490 patients (8.4%). Post-discharge surgical complications accounted for 102 readmissions (1.8%), of which 81 occurred within 1 month and 21 within 3 months.

**Fig. 2:**
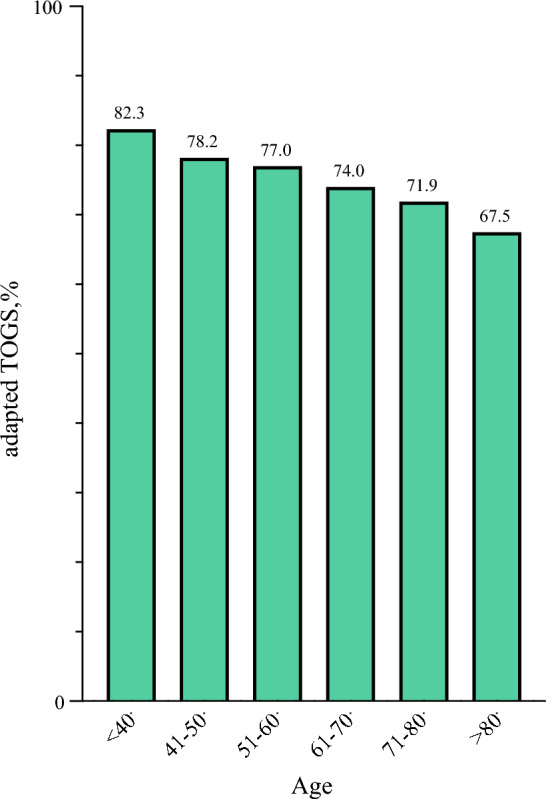
Age-stratified Textbook Outcome in Gastric Surgery (TOGS) achievement

The achievement of TOGS declined with age, from 82.3% in patients aged < 40 years to 67.5% in those aged >80 years. Although individual parameters such as negative resection margins (> 98.3%) and absence of early mortality (>99.3%) remained consistently high across all age groups, older patients showed reduced composite TOGS. (Fig. 2; Supplementary 1).

### Trends over time

**Fig. 3:**
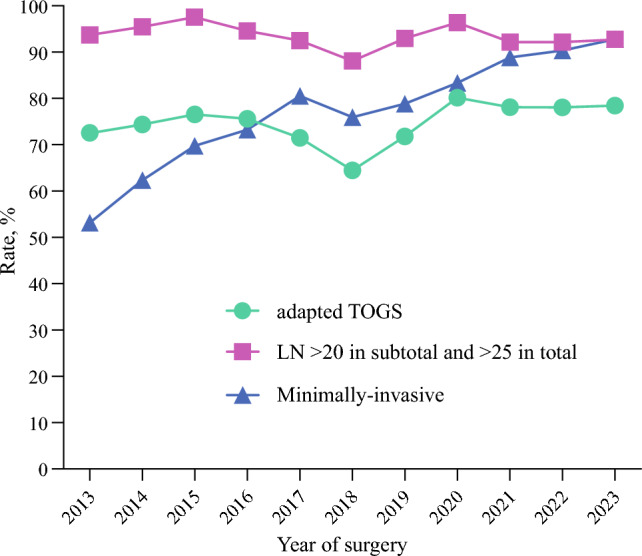
Textbook Outcome in Gastric Surgery (TOGS) achievement over time. LN, lymph nodes

Figure 3 shows the longitudinal trends in surgical indicators between 2013 and 2023. The rate of surgeries meeting the TOGS criteria remained largely stable throughout the study period, ranging from 64.5 to 80.2%. The proportion of patients with adequate lymph node yield (> 20 in subtotal and > 25 in total gastrectomy as defined by TOGS criteria) was consistently high across all years, exceeding 90% in most periods and peaking at 97.6% in 2015. Compliance with adequate lymph node retrieval was lower among male patients, older age groups, total gastrectomies, advanced disease (*P* < 0.001), and patients with higher BMI (*P* = 0.023) but was not influenced by surgical approach. Notably, the proportion of surgeries fulfilling TOGS criteria remained stable over the study period and closely reflected adequacy of lymph node yield (*P* < 0.001).

In contrast, the use of minimally invasive surgery increased steadily, rising from 68.5% before 2018 to 86.5% from 2019 onward, with no cases of early postoperative mortality observed in the latter period (Supplementary 2).

In multivariable analysis, more recent surgery (2019–2023), female sex, age ≤ 65 years, distal gastrectomy, surgical approach, and early pT stages were strong independent predictors of TOGS achievement. Conversely, BMI and lymph node metastases were not significant after adjustment (Table 3). Moreover, the rate of reintervention significantly decreased in recent years (*P* < 0.001). Reintervention was more likely in males, after open surgery, in advanced disease (*P* < 0.001), or after total gastrectomy (*P* = 0.019) but was not influenced by age or BMI. Similarly, advanced stage (*P* < 0.001) and total gastrectomy (*P* = 0.005) predicted a higher likelihood of hospital readmission.

**Table 3:** Predictive factors associated with adapted Textbook Outcome in Gastric Surgery (TOGS) attainment

Variable	Univariable		Multivariable	
	OR (95% Cl)	*P*-value	OR (95% Cl)	*P*-value
*Baseline*				
Surgery year 2019–2023	1.26 (1.11–1.42)	**<0.001**	1.16 (1.02–1.31)	**0.024**
Female sex	1.43 (1.25–1.63)	**<0.001**	1.38 (1.21–1.58)	**<0.001**
Age, ≤65 years	1.33 (1.18–1.50)	**<0.001**	1.36 (1.21–1.58)	**<0.001**
BMI ≤25 kg/m^2^	1.02 (0.90–1.15)	0.788	–	–
*Surgery*				
Distal gastrectomy	1.86 (1.63–2.12)	**<0.001**	1.50 (1.31–1.74)	**<0.001**
Minimally invasive approach	1.81 (1.58–2.07)	**<0.001**	1.51 (1.29–1.77)	**<0.001**
*Tumor*				
pN stage N0	1.41 (1.24–1.59)	**<0.001**	0.99 (0.85–1.15)	0.871
pT stage T1–2	1.74 (1.53–1.97)	**<0.001**	1.30 (1.09–1.54)	**0.003**

Bold indicates significant results

BMI Body mass index, CI Confidence interval, OR Odds ratio, Ref Reference

### Length of Hospital Stay

Hospital stay significantly differed between patients who achieved the adapted TOGS and others (*P* < 0.001) as shown in Table 2. A simple correlation showed an inverse correlation between adapted TOGS achievement over years and hospital stay (*r* = − 0.711; *P* = 0.014), even after adjustment for age and BMI (*r* = − 0.658; *P* = 0.028).

### Survival Outcomes

**Fig. 4:**
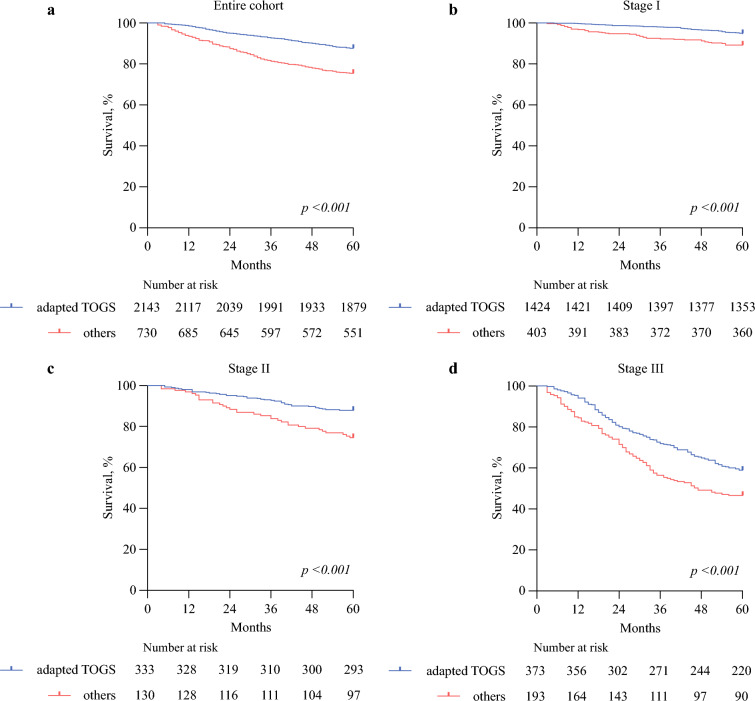
Kaplan–Meier curves comparing overall survival in entire cohort and according to pathological tumor stages

Median survival in a subgroup of 2886 patients operated on between 2013 and 2017 was 74 months. Survival data are shown in Fig. 4.

Patients reaching the DUCA definition of TO had a 5-year OS rate of 87.6%, versus 75.6% for patients with non TO (ratio 0.96 [95% confidence interval {CI} 0.82–1.14], *P* < 0.001).

Patients with adapted TOGS had a 5-year OS rate of 87.6% versus 74.3% (ratio 0.97 [95% CI 0.82–1.15], *P* < 0.001; Fig. 4a), 94.9% versus 89.3% for patients with stage I tumor (*P* < 0.001; Fig. 4b), 88.0% versus 74.6% (*P* < 0.001; Fig. 4c) for those with stage II tumor, and 59.0% versus 46.6% (*P* < 0.001; Fig. 4d) for those with stage III tumor. Multivariable Cox regression analysis confirmed the adapted TOGS as an independent prognostic factor (ratio 0.63 [95% CI 0.53–0.76], *P* < 0.001) (Supplementary 3).

Among the 6.8% of patients enrolled in TO but excluded from TOGS (delta group), survival analysis showed a substantially lower survival trajectory, with a median of 61 months and a 5-year OS of 72.5% (Supplementary 4).

## Discussion

International guidelines promote the establishment of high-quality cancer services and offer a comprehensive description of the essential requirements for effective care of patients with GC.^15–17^ Common metrics, including surgical mortality and complication rates, have traditionally been used to measure hospital performance.^18^ However, these single outcomes have low event rates, limiting statistical power, and do not describe the entirety of the perioperative process.^5^ Similarly, the Enhanced Recovery After Surgery protocols demonstrate clear benefits in postoperative recovery,^19^ but their universal applicability remains limited because of patient heterogeneity and resource demands.^20^

In 2017, the Dutch DUCA group introduced a TO for gastrointestinal malignancies as a noninterventional benchmark of quality of care. The group reported a positive association between hospital volume and the likelihood of achieving TO, ranging from 11.4% in low-volume centers to 52.4% in high-volume centers.^14^

Nevertheless, most published studies have been conducted in Western countries, where the proportion of obese patients with advanced tumors requiring neoadjuvant treatment and the incidence of open surgery are considerably higher than in East Asia.^8,21^ In this context, the rate of TO achievement is lower in Western patients (up to 59%) than in Eastern (up to 84%),^22^ a key barrier remaining the number of lymph nodes retrieved (at least 15) in the resected specimen.

More recently, a GC-specific definition of TOGS emerged from a consensus among members of GIRCG Italian group, aiming to facilitate the comparison of outcomes across centers worldwide.^13^ The new TOGS justified the exclusion of selected items from the original definition, as they were deemed redundant or arbitrary. Moreover, TOGS recommends the retrieval of at least 20 lymph nodes for subtotal gastrectomy and 25 lymph nodes for total gastrectomy.^23^ We previously described that the number of examined lymph nodes increased with advancing tumor stage and that survival outcomes were significantly improved after harvesting 30 or more lymph nodes (all stages hazard ratio [HR] 0.56 [95% CI 0.39–0.82], *P* = 0.002; stage I HR 0.62 [95% CI 0.37–1.06], *P* = 0.080; stage II–III HR 0.48 [95% CI 0.29–0.82], *P* = 0.007).^24^ However, such a high threshold may limit applicability in lower-volume centers or for frail patients and early lesions, where a more limited lymphadenectomy is often indicated.^25^

In the present study 74.7% of patients achieved TOGS adapted for Eastern patients, limited to surgical parameters. Although this differs from the original TOGS definition,^13^ the exclusion of the oncological item was necessary, as local guidelines diverge from those commonly adopted in Western settings. Further validation of this version of TOGS is being promoted in referral East Asian centers.

The adequate number of lymph nodes collected was confirmed at 93.7%, similar to the 87.6% described in the GIRCG study. Notably, the proportion of lymph nodes removed remained consistently high throughout all years, exceeding 90% in most periods, which is a close reflection of the TOGS success and indicates that these metrics have been achieved in parallel. Our trajectories slightly differed from those of the multicenter US centers, which have improved the rate of patients with at least 16 lymph nodes removed (from 40.4% in 2000–2004 to 87.2% in 2015–2020) and the rate of patients with a TO (from 14.9% in 2000–2004 to 53.9% in 2015–2020).^12^

On the other hand, the TOGS rate has risen alongside the growing adoption of minimally invasive surgery. Between 2013 and 2018, TOGS was achieved in 72.9% of cases, of which 68.5% were performed via laparoscopic or robotic approaches, increasing to 77.2% TOGS in the more recent period with minimally invasive techniques accounting for 86.5% of procedures (odds ratio [OR] 1.51 [95% CI 1.29–1.77], *P* < 0.001). This trend may reflect the results of the KLASS-02 trial, which demonstrated the noninferiority of the laparoscopic approach compared with open surgery in 1011 advanced cases. In particular, the Korean trial showed a morbidity rate significantly lower after laparoscopic than after open gastrectomy (16.6% vs. 24.1%; *P* = 0.003), whereas early mortality was similar between the two groups (laparoscopic 0.4% vs. open 0.6%; *P* = 0.682),^26^ and no significant differences were observed in 5-year OS (*P* = 0.300) or relapse-free survival (*P* = 0.658),^27^ supporting the safety of minimally invasive surgery even for advanced GC.^28^

Another important consideration is the variation in TOGS according to patients and tumor variables. An analysis of the Spanish EURECCA registry showed that age was an independent predictor of failure to achieve a TO in GC surgery, with patients aged 65–74 years having lower odds (OR 0.53 [95% CI 0.39–0.73], *P* < 0.001), and the effect was even more pronounced in the elderly (OR 0.34 [95% CI 0.24–0.48], *P* < 0.001).^29^ Similarly, we described a progressive decline in the achievement of TOGS with increasing age, empathizing a strong threshold effect starting after 65 years (OR 1.36 [95% Cl 1.21–1.58], *P* < 0.001). However, patients achieving TOGS experienced significantly shorter hospital stays, even after adjustment for age and BMI, underscoring its robustness as a marker of optimal use of hospital resources. Our data suggest that optimizing prehabilitation, ensuring nutritional support, and early recognition of potential red flags may further enhance perioperative outcomes in high-risk patients less likely to achieve TOGS (e.g., elderly, males, open, total gastrectomy, advanced disease).^30,31^

Notably, TOGS was achieved in 77.9% of patients with stage I compared with 68.8% of patients with stage II–III tumors. Advanced cases were associated with higher re-intervention (15.3% vs. 8.2%) and hospital readmission rates (14.6% vs. 7.9%), although the completeness in lymphadenectomy (96.2% vs. 92.1%) and negative resection margins, routinely verified intraoperatively after stomach transection,^32,33^ remained similar (98.6% vs. 99.9%). On further examination of the results, the pN parameter did not demonstrate a significant association with the achievement of TOGS, which may be explained by the consistently high number of lymph nodes retrieved regardless of the preoperative clinical stage (OR 0.99 [95% CI 0.85–1.15], *P* = 0.871). These findings are consistent with those reported by the GIRCG study. Conversely, the pT parameter emerged as a determinant factor, serving as an indirect measure of tumor size and reflecting both increased technical difficulty of resection and, when performed, an inadequate or absent response to neoadjuvant chemotherapy.

Lastly, as in previous studies, our survival data showed significant differences in 5-year OS between the TOGS and non-TOGS groups (87.6% vs. 75.6%), irrespective of the stage of tumor.^24,34^ Additionally, TOGS more effectively excludes patients with poorer prognosis than does the original TO, suggesting a higher positive predictive value in selectively identifying individuals with favorable survival outcomes in stage I (*P* = 0.002) and stages II–III (*P* < 0.001).

Our study has inherent limitations due to its single-center design, which may limit the universality of its findings. Patients were enrolled in a high-volume hospital, which may not fully reflect outcomes in the real world. Furthermore, the algorithm under investigation was developed in the West, and direct comparisons with Eastern patients should be made cautiously because of the geographical variability in tumor characteristics and perioperative management.

In conclusion, this study demonstrated concordance in perioperative outcomes between our cohort and those reported from other Eastern specialty centers. The new TOGS selectively identified patients with a potentially better prognosis, thereby supporting its role in empowering patient decision-making.

## Supplementary Information

Below is the link to the electronic supplementary material.

Supplementary file1 (DOCX 264 KB)

## References

[1] Thrift AP, Wenker TN, El-Serag HB. Global burden of gastric cancer: epidemiological trends, risk factors, screening and prevention. *Nat Rev Clin Oncol*. 2023;20(5):338–49. 10.1038/s41571-023-00747-0. 36959359 10.1038/s41571-023-00747-0

[2] Leijonmarck W, Mattsson F, Lagergren J. Survival among patients cured from gastric adenocarcinoma compared to the background population. *Gastric Cancer*. 2024;27(6):1180–8. 10.1007/s10120-024-01545-y. 39230776 10.1007/s10120-024-01545-y PMC11513714

[3] Park SH, Kang MJ, Yun EH, Jung KW. Epidemiology of gastric cancer in Korea: trends in incidence and survival based on Korea central cancer registry data (1999–2019). *J Gastric Cancer*. 2022;22(3):160. 10.5230/jgc.2022.22.e21. 35938363 10.5230/jgc.2022.22.e21 PMC9359882

[4] Choi Y, Kim N, Kim JH, et al. Survival rates of patients with gastric cancer according to age and sex: a large-scale study using data from 14,739 patients. *Cancer Res Treat*. 2026;58(1):252–63. 10.4143/crt.2025.149. 40241577 10.4143/crt.2025.149 PMC12800952

[5] Marano L, Verre L, Carbone L, et al. Current trends in volume and surgical outcomes in gastric cancer. *J Clin Med*. 2023;12(7):2708. 10.3390/jcm12072708. 37048791 10.3390/jcm12072708 PMC10094776

[6] Cho YS, Berlth F, Kim J, et al. Clinical outcomes of robotic and laparoscopic gastrectomy using propensity score matching method: Data of 5-year period in a Korean high-volume gastric cancer center. *Eur J Surg Oncol*. 2025;51(8):110014. 10.1016/j.ejso.2025.110014. 40203541 10.1016/j.ejso.2025.110014

[7] Engborg J, Winbladh A, Lindblad M, Hedberg J. Centralization of gastric cancer surgery: impact on treatment strategies and survival—a national population-based cohort study. *J Gastrointest Surg*. 2025;29(1):101879. 10.1016/j.gassur.2024.101879. 39510159 10.1016/j.gassur.2024.101879

[8] Yamamoto M, Rashid OM, Wong J. Surgical management of gastric cancer: the East versus West perspective. *J Gastrointest Oncol*. 2015;6(1):79–88. 10.3978/j.issn.2078-6891.2014.097. 25642341 10.3978/j.issn.2078-6891.2014.097 PMC4294827

[9] van der Werf LR, Wijnhoven BPL, Fransen LFC, et al. A national cohort study evaluating the association between short-term outcomes and long-term survival after esophageal and gastric cancer surgery. *Ann Surg*. 2019;270(5):868–76. 10.1097/SLA.0000000000003520. 31634182 10.1097/SLA.0000000000003520

[10] Oh SG, Lee S, Seong BO, et al. Textbook outcome of delta-shaped anastomosis in minimally invasive distal gastrectomy for gastric cancer in 4,505 consecutive patients. *J Gastric Cancer*. 2024;24(3):341. 10.5230/jgc.2024.24.e29. 38960892 10.5230/jgc.2024.24.e29 PMC11224722

[11] Velayudham GK, Dermanis A, Kamarajah SK, Griffiths EA. Predictors of textbook outcome following oesophagogastric cancer surgery. *Dis Esophagus*. 2024;37(7):doae023. 10.1093/dote/doae023. 38525934 10.1093/dote/doae023 PMC11220663

[12] Spolverato G, Paro A, Capelli G, et al. Surgical treatment of gastric adenocarcinoma: are we achieving textbook oncologic outcomes for our patients? *J Surg Oncol*. 2022;125(4):621–30. 10.1002/jso.26778. 34964983 10.1002/jso.26778

[13] Marrelli D, Carbone L, Piccioni SA, et al. Textbook outcome in gastric cancer surgery: a multicenter cohort study and proposal for a new specific index (TOGS). *Gastric Cancer*. 2026;29(2):452–64. 10.1007/s10120-025-01710-x. 41511638 10.1007/s10120-025-01710-x

[14] Busweiler LAD, Schouwenburg MG, van Berge Henegouwen MI, et al. Textbook outcome as a composite measure in oesophagogastric cancer surgery. *Br J Surg*. 2017;104(6):742–50. 10.1002/bjs.10486. 28240357 10.1002/bjs.10486

[15] Stroobant EE, Kong SH, Bencivenga M, et al. Korea, Japan, Europe, and the United States: Why are guidelines for gastric cancer different? *Gastric Cancer*. 2025;28(4):559–68. 10.1007/s10120-025-01613-x. 40240698 10.1007/s10120-025-01613-x

[16] Japanese Gastric Cancer Association. Japanese gastric cancer treatment guidelines 2014 (ver. 4). *Gastric Cancer*. 2017;20(1):1–19. 10.1007/s10120-016-0622-4. 10.1007/s10120-016-0622-4 PMC5215069 27342689

[17] Kim IH, Kang SJ, Choi W, et al. Korean practice guidelines for gastric cancer 2024: an evidence-based, multidisciplinary approach (update of 2022 guideline). *J Gastric Cancer*. 2025;25(1):5. 10.5230/jgc.2025.25.e11. 39822170 10.5230/jgc.2025.25.e11 PMC11739648

[18] Sheetz KH, Ibrahim AM, Nathan H, Dimick JB. Variation in surgical outcomes across networks of the highest-rated US hospitals. *JAMA Surg*. 2019;154(6):510. 10.1001/jamasurg.2019.0090. 30865220 10.1001/jamasurg.2019.0090 PMC6583390

[19] Kehlet H. Multimodal approach to control postoperative pathophysiology and rehabilitation. *Br J Anaesth*. 1997;78(5):606–17. 10.1093/bja/78.5.606. 9175983 10.1093/bja/78.5.606

[20] Lof S, Benedetti Cacciaguerra A, Aljarrah R, et al. Implementation of enhanced recovery after surgery for pancreatoduodenectomy increases the proportion of patients achieving textbook outcome: a retrospective cohort study. *Pancreatology*. 2020;20(5):976–83. 10.1016/j.pan.2020.05.018. 32600854 10.1016/j.pan.2020.05.018

[21] Bickenbach K, Strong VE. Comparisons of Gastric Cancer Treatments: east versus west. *J Gastric Cancer*. 2012;12(2):55. 10.5230/jgc.2012.12.2.55. 22792517 10.5230/jgc.2012.12.2.55 PMC3392325

[22] Carbonell-Morote S, Yang HK, Lacueva J, et al. Textbook outcome in oncological gastric surgery: a systematic review and call for an international consensus. *World J Surg Oncol*. 2023;21(1):288. 10.1186/s12957-023-03166-8. 37697286 10.1186/s12957-023-03166-8 PMC10496160

[23] Verlato G, Roviello F, Marchet A, et al. Indexes of surgical quality in gastric cancer surgery: experience of an Italian network. *Ann Surg Oncol*. 2009;16(3):594–602. 10.1245/s10434-008-0271-x. 19118437 10.1245/s10434-008-0271-x

[24] Cho YS, Kim S, Kim J, et al. Textbook outcome of gastric cancer surgery and lymph node evaluation as its parameter to improve long-term survival. *Sci Rep*. 2025;15(1):34159. 10.1038/s41598-025-14971-4. 41034377 10.1038/s41598-025-14971-4 PMC12489070

[25] Wakahara T, Ueno N, Maeda T, et al. Impact of gastric cancer surgery in elderly patients. *Oncology*. 2018;94(2):79–84. 10.1159/000481404. 29045948 10.1159/000481404

[26] Lee HJ, Hyung WJ, Yang HK, et al. Short-term outcomes of a multicenter randomized controlled trial comparing laparoscopic distal gastrectomy with D2 lymphadenectomy to open distal gastrectomy for locally advanced gastric Cancer (KLASS-02-RCT). *Ann Surg*. 2019;270(6):983–91. 10.1097/SLA.0000000000003217. 30829698 10.1097/SLA.0000000000003217

[27] Son SY, Hur H, Hyung WJ, et al. Laparoscopic versus open distal gastrectomy for locally advanced gastric cancer. *JAMA Surg*. 2022;157(10):879. 10.1001/jamasurg.2022.2749. 35857305 10.1001/jamasurg.2022.2749 PMC9301593

[28] Marrelli D, Carbone L, Poto GE, et al. Minimally invasive lymphadenectomy for gastric cancer: Could the robotic approach provide any benefits than laparoscopy? *World J Gastrointest Oncol*. 2025;17(6):104015. 10.4251/wjgo.v17.i6.104015. 40547156 10.4251/wjgo.v17.i6.104015 PMC12179926

[29] Dal Cero M, Román M, Grande L, et al. Textbook outcome and survival after gastric cancer resection with curative intent: a population-based analysis. *Eur J Surg Oncol*. 2022;48(4):768–75. 10.1016/j.ejso.2021.10.025. 34753620 10.1016/j.ejso.2021.10.025

[30] Marano L, Marmorino F, Desideri I, et al. Clinical nutrition in surgical oncology: young AIOM-AIRO-SICO multidisciplinary national survey on behalf of NutriOnc research group. *Front Nutr*. 2023;10:1045022. 10.3389/fnut.2023.1045022. 37125048 10.3389/fnut.2023.1045022 PMC10140427

[31] Yoon SH, Ju JW, Lee HJ, et al. Development of the Korean enhanced recovery after surgery audit program. *Sci Rep*. 2025;15(1):27409. 10.1038/s41598-025-10622-w. 40721595 10.1038/s41598-025-10622-w PMC12304143

[32] Berlth F, Kim WH, Choi JH, et al. Prognostic impact of frozen section investigation and extent of proximal safety margin in gastric cancer resection. *Ann Surg*. 2020;272(5):871–8. 10.1097/SLA.0000000000004266. 32833759 10.1097/SLA.0000000000004266

[33] Lin X, Tan C, Wu W, et al. Association between textbook outcome and long-term survival among patients undergoing curative-intent resection of gastric cancer. *Surgery*. 2024;176(5):1402–11. 10.1016/j.surg.2024.07.022. 39181724 10.1016/j.surg.2024.07.022

[34] Chen JY, Lin GT, Chen QY, et al. Textbook outcome, chemotherapy compliance, and prognosis after radical gastrectomy for gastric cancer: A large sample analysis. *Eur J Surg Oncol*. 2022;48(10):2141–8. 10.1016/j.ejso.2022.05.025. 35780034 10.1016/j.ejso.2022.05.025

