# Supplementary material for: Validation of Textbook Outcome in Gastric Surgery (TOGS) for Primary Gastric Cancer in an Eastern High-Volume Center
Source: Ann Surg Oncol. 2026 Apr 3;33(8):7283–92. doi: 10.1245/s10434-026-19522-3 (PMC13337739; doi:10.1245/s10434-026-19522-3)

**Validation of Textbook Outcome in Gastric Surgery (TOGS) for primary gastric cancer in an Eastern high volume center**

**Authors:**

Ludovico Carbone MD^1,2^ (co-first), Yo-Seok Cho MD,MS^1,3^ (co-first), Min Kyu Kang MD,MBBS^1,3^, Kyoyoung Park MD^1,3^, Chungyoon Kim MD,MS^1,3^, Sa-Hong Kim MD,MS^1,3^, Jeesun Kim MD,PhD^1,3^, Nina Rebecca Kalaw MD^1,4^, Yoonjin Kwak MD,Ph^5,6,7^, Hye Seung Lee MD,PhD^5,6,7^, Seong-Ho Kong MD,MS^1,3,7^, Do Joong Park MD,PhD^1,3,7^, Daniele Marrelli MD^2,8^, Han-Kwang Yang MD,PhD^1,3,7^, Franco Roviello MD^2,8^, Hyuk-Joon Lee MD,PhD^1,3,7^

**Supplementary 1.** Age-stratified achievement of TOGS parameters.

|  | <40  (n=231) | 41-50  (n=693) | 51-60  (n=1,463) | 61-70  (n=1,816) | 71-80  (n=1,311) | >81  (n=292) |
| --- | --- | --- | --- | --- | --- | --- |
| TOGS | 190 (82.3) | 542 (78.2) | 1,127 (77.0) | 1,343 (74.0) | 943 (71.9) | 197 (67.5) |
| no intraoperative complication | 226 (97.8) | 690 (99.6) | 1,439 (98.4) | 1,795 (98.8) | 1,294 (98.7) | 285 (97.6) |
| negative resection margins | 229 (99.1) | 691 (99.7) | 1,457 (99.6) | 1,806 (99.4) | 1,305 (99.7) | 287 (98.3) |
| adequate lymphadenectomy | 225 (97.4) | 659 (95.1) | 1,394 (95.3) | 1,705 (93.9) | 1,188 (90.6) | 270 (92.5) |
| no re-intervention | 213 (92.2) | 631 (91.1) | 1,316 (90.0) | 1,597 (87.9) | 1,171 (89.3) | 261 (89.7) |
| no unplanned ICU | 230 (99.6) | 689 (99.4) | 1,446 (98.8) | 1,794 (98.8) | 1,272 (97.0) | 282 (96.6) |
| no unplanned 90-day hospital readmission | 209 (90.5) | 621 (89.6) | 1,333 (91.1) | 1,619 (89.2) | 1,188 (90.6) | 244 (83.6) |
| no 90-day mortality | 230 (99.6) | 690 (99.6) | 1,462 (99.9) | 1,814 (99.9) | 1,307 (99.7) | 290 (99.3) |
| Total Survival (median mo.; 5y) | Ref; 85.5 | 80; 86.3 | 72; 89.4 | 77; 85.3 | 69; 77.7 | 71; 61.5 |

Acronym details: TOGS Textbook Outcome in Gastric Surgery; ICU intensive-care unit.

**Supplementary 2.** Achievement of TOGS and its individual parameters over time.

|  | 2013-2018  (n=3,351) | 2019-2023  (n=2,455) |
| --- | --- | --- |
| adapted TOGS | 2,443 (72.9) | 1,895 (77.2) |
| no intraoperative complication | 3,305 (98.6) | 2,424 (98.7) |
| negative resection margins | 3,334 (99.5) | 2,441 (99.4) |
| adequate lymphadenectomy | 3,148 (93.9) | 2,293 (93.4) |
| no re-intervention | 2,915 (87.0) | 2,274 (92.6) |
| no unplanned ICU | 3,284 (98.0) | 2,429 (98.9) |
| no 90-day unplanned hospital readmission | 2,998 (89.5) | 2,216 (90.3) |
| no 90-day mortality | 3,338 (99.6) | 2,455 (100.0) |
| Minimally-invasive approach | 2,297 (68.5) | 2,123 (86.5) |

Acronym details: TOGS Textbook Outcome in Gastric Surgery; ICU intensive-care unit.

**Supplementary 3.** Multivariable Cox proportional hazards model.

| Variable | Multivariable | |
| --- | --- | --- |
|  | HR (95% Cl) | P |
| Baseline | | |
| Adapted TOGS | 0.63 (0.53 – 0.76) | **<0.001** |
| Gender Female | 1.05 (0.88 – 1.25) | 0.603 |
| Age ≤ 65 years | 0.59 (0.49 – 0.70) | **<0.001** |
| BMI ≤ 25 kg/m^2^ | 1.17 (0.96 – 1.42) | 0.102 |
| Surgery | | |
| Distal Gastrectomy | 0.97 (0.80 – 1.17) | 0.723 |
| Minimally-invasive approach | 0.77 (0.62 – 0.95) | **0.014** |
| Tumour | | |
| pN0 | 0.47 (0.38 – 0.57) | **<0.001** |
| pT1-2 | 0.42 (0.34 – 0.52) | **<0.001** |

Acronym details: HR Hazard ratio; BMI body mass index.

**Supplementary 4.** Survival differences between patients classified as TO (DUCA), adapted TOGS (GIRCG), and those achieving TO but excluded from adapted TOGS (delta TO–TOGS), in the whole cohort, stage I, and stage II–III disease.


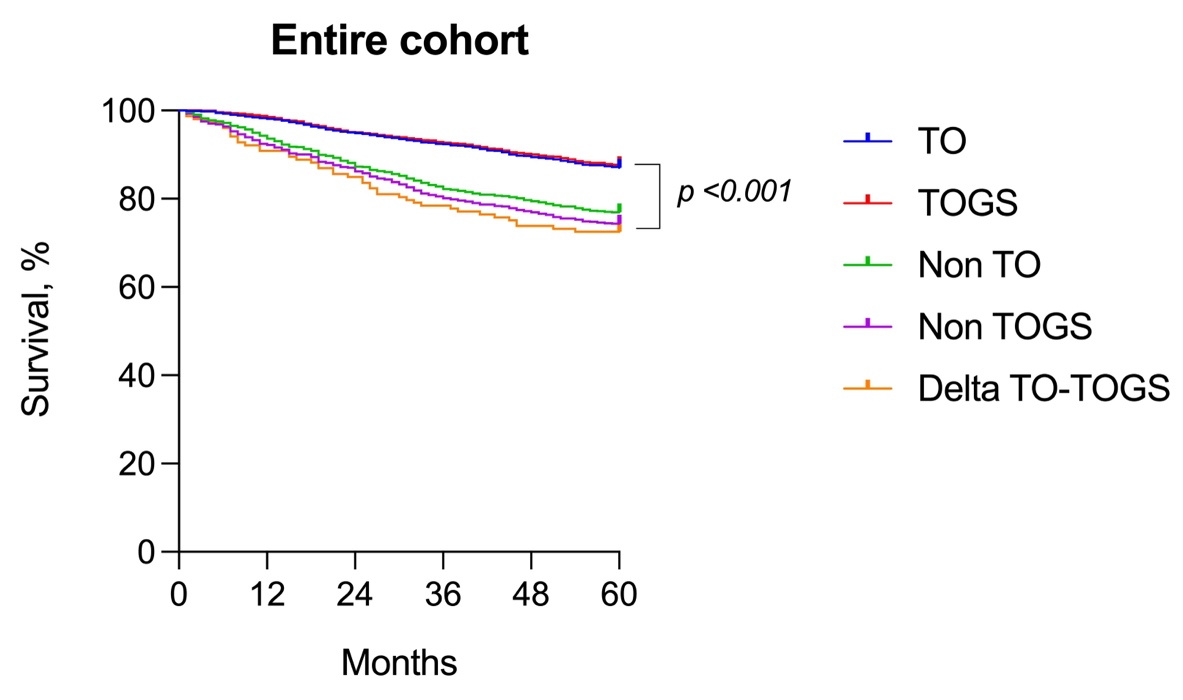


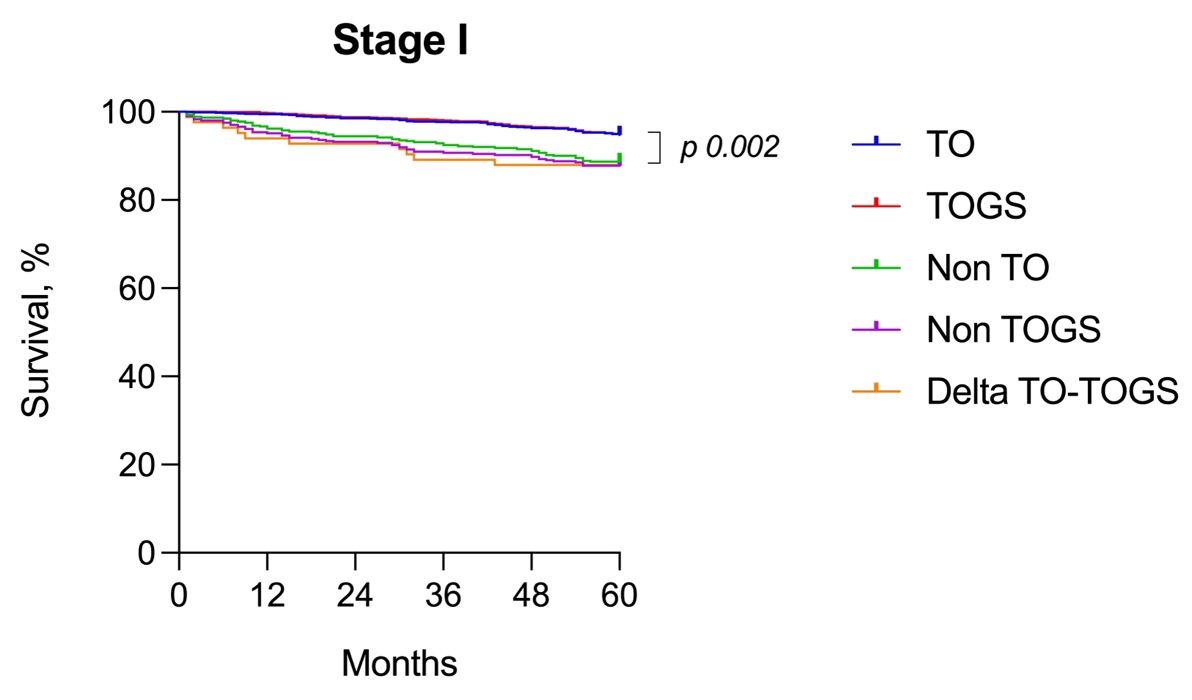


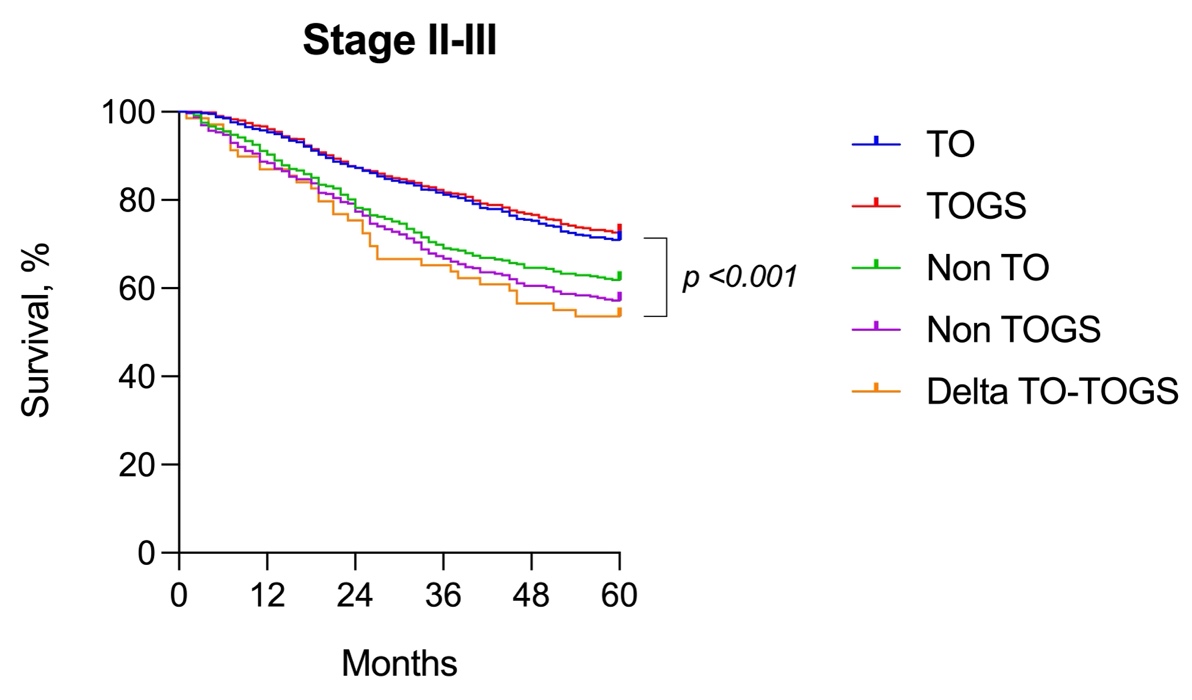

Supplement: Supplementary file 1 — Supplementary file1 (DOCX 264 KB) [file 10434_2026_19522_MOESM1_ESM.docx]
